# Inflammatory Bowel Disease, Gastrointestinal Graft-Versus-Host Disease and Immune Checkpoint Inhibitors Induced Colitis: Similar Diseases to Treat with Fecal Microbiota Transplantation

**DOI:** 10.3390/nu17233788

**Published:** 2025-12-03

**Authors:** Giuseppe Biscaglia, Annamaria Gentile, Paola Parente, Annamaria Calvo, Rosanna Fontana, Antonio Continisio, Anna Laura Pia Di Brina, Davide Ciardiello, Gillian McIlwain, Anna Latiano, Francesco Perri, Orazio Palmieri

**Affiliations:** 1 Division of Gastroenterology, Fondazione IRCCS Casa Sollievo della Sofferenza, 71013 San Giovanni Rotondo, Italyo.palmieri@operapadrepio.it (O.P.); 2 Pathology Unit, Fondazione IRCCS Casa Sollievo della Sofferenza, 71013 San Giovanni Rotondo, Italy; 3 Microbiology Unit, Fondazione IRCCS Casa Sollievo della Sofferenza, 71013 San Giovanni Rotondo, Italy; 4 Section of Gastroenterology, Department of Emergency and Organ Transplantation, University “Aldo Moro” Bari, 70121 Bari, Italy; 5 Division of Gastrointestinal Medical Oncology and Neuroendocrine Tumors, European Institute of Oncology (IEO), IRCCS, 20141 Milan, Italy; 6 Medical School, The University of Queensland, Herston, QLD 4006, Australia

**Keywords:** FMT, IBD, CD, UC, GVHD, ICI-iC

## Abstract

Fecal microbiota transplantation (FMT) is a therapeutic strategy designed to modify and enrich the recipient’s gut microbiota by administering processed donor stool, with the goal of treating dysbiosis and related conditions. In 2013, the United States Food and Drug Administration (FDA) approved FMT for recurrent *Clostridioides difficile* infection (rCDI). Since then, its use has been proposed and investigated in several other disorders characterized by gut microbiota imbalance and altered host–microbiota interactions, including inflammatory bowel disease (IBD), immune checkpoint inhibitor-induced colitis (ICI-iC), and gastrointestinal graft-versus-host disease (GI-GVHD). This review aims to highlight the commonalities among these conditions, the pathophysiological mechanisms that support the rationale for FMT, and emerging evidence from clinical studies. Although available studies are heterogeneous, FMT is a rapidly evolving field of research with promising potential to treat IBD and improve outcomes following oncological immunotherapy and allogenic stem cell transplantation. With further validation, FMT could become an important approach in managing immune-mediated gastrointestinal diseases.

## 1. Introduction

Fecal microbiota transplantation (FMT) involves the collection of stool from a healthy donor, which is then processed and transferred to a recipient with a quantitative or qualitative alteration of the gut microbiota, aiming to restore microbial composition and functions. The first randomized controlled trial of FMT was conducted in 2013, in which fresh donor stool was delivered via duodenal infusion to treat recurrent *Clostridioides difficile* infection (rCDI) [1].

In the same year, the U.S. Food and Drug Administration (FDA) approved FMT for rCDI. Over the past decade, there has been significant progress in managing *C. difficile* infection, most notably through the introduction of bezlotoxumab, a fully human monoclonal antibody targeting toxin B, which has been shown to reduce recurrence rates when added to standard-of-care antibiotic therapy [2].

More recently, the FDA has approved two standardized, self-administered FMT products, Rebyota, a single-dose enema, and Vowst, an oral capsule formulation, both indicated for rCDI [3]. FMT can be delivered through multiple routes, including feeding tubes (nasoduodenal or nasojejunal), enemas, endoscopic infusion during colonoscopy or proctosigmoidoscopy, and oral capsules, each with specific advantages and limitations.

A large-scale analysis of cases from 2000 to 2020 reported an adverse event rate of 19%, mostly mild and self-limiting events (e.g., nausea, bloating, vomiting, diarrhea), with procedure-related mortality estimated at 0.02% [4].

**Figure 1:**
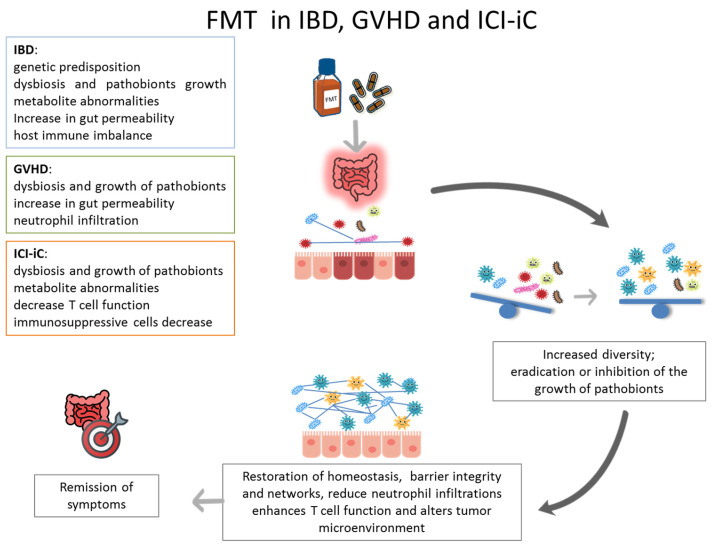
The diagram illustrates the function of the FMT in the treatment of IBD, GVHD and ICI-iC, with the aim of restoring intestinal homeostasis and remission of symptoms.

Beyond rCDI, FMT is increasingly being investigated for other gastrointestinal conditions, including inflammatory bowel disease (IBD), immune checkpoint inhibitor-induced colitis (ICI-iC), and gastrointestinal graft-versus-host disease (GI-GVHD) [5]. These disorders share clinical, endoscopic, histological and therapeutic features, as well as a common pathogenic hallmark. It has been hypothesized that this is due to a dysregulation of tolerance between the intestinal microbiota and the host. Growing evidence suggests that dysbiosis is a key driver of their pathogenesis (Figure 1). Unlike current therapies, which mainly act on extra-luminal targets (e.g., immunomodulators), FMT acts directly within the intestinal lumen to re-establish microbial balance, thereby supporting host health and intestinal homeostasis.

## 2. Methods

For this narrative review, a literature search was conducted across PubMed/MEDLINE, Embase, and the Cochrane Library databases to identify relevant studies published from 2015 up to September 2025. The search strategy incorporated MeSH terms including “Inflammatory Bowel Disease”, “Crohn’s Disease”, “Ulcerative Colitis”, “Gastrointestinal Graft-Versus-Host Disease”, and “Immune Checkpoint Inhibitor–Induced Colitis”, combined with the Boolean operator AND to additional MeSH terms such as “FMT” and “Fecal Microbiota Transplantation”. This approach was designed to capture all studies investigating FMT-based interventions in intestinal immune-mediated inflammatory conditions.

Eligible studies included those enrolling patients with IBD, UC, CD, GI-GVHD, or ICI-iC, irrespective of study design (randomized, observational, or case series), number of participating centers, or age group (adult). No restrictions were applied regarding sample size, route of FMT administration, donor selection strategy, or treatment protocol. Only articles published in English were included (Table 1).

As an example, for CD, one of the Boolean search strings strategies employed in PubMed was (Crohn disease) AND (FMT), article type Clinical Trial or Randomized Controlled Trial, to identify articles at (https://pubmed.ncbi.nlm.nih.gov/?term=crohn+disease+and+fmt&filter=dates.2020-2025&filter=pubt.clinicaltrial&filter=pubt.randomizedcontrolledtrial&sort=date, 25 August 2025). This strategy initially retrieved 8 records published in the last five years (from 1 January 2020 to August 2025).

After the removal of duplicates, three reviewers (G.B., A.L., and G.C.) independently screened titles and abstracts according to predefined inclusion and exclusion criteria. Full texts of potentially eligible studies were retrieved and assessed for final inclusion. Any discrepancies were resolved by consensus or, when necessary, through consultation with a fourth independent and experienced reviewer (O.P.).

## 3. FMT in Inflammatory Bowel Disease

IBD is a group of chronic, immune-mediated disorders of the gastrointestinal tract, comprising two main entities: Crohn’s disease (CD) [31] and ulcerative colitis (UC) [32].

Crohn’s disease can affect any segment of the gastrointestinal tract, from the mouth to the anus. Inflammation is typically transmural, may follow a penetrating pattern, and is characteristically discontinuous, producing so-called “skip lesions” [31]. Ulcerative colitis predominately involves the colonic mucosa, with continuous inflammation of variable extent from the distal to the proximal colon.

IBD arises from a complex interplay of genetic susceptibility, environmental exposures, and immune dysregulation. These environmental factors facilitate the dysregulation of the immune system by triggering an abnormal response that culminates in persistent bowel inflammation [33].

The histological diagnosis of IBD is based on the evaluation of ‘minimal lesions’ present in the bowel: changes in glandular component (glandular loss or atrophy; glandular architectural changes or distortion); changes in the cellular component of the glands (mucodepletion, muciparous hyperplasia, Paneth cell metaplasia in the left colic segments, sigma and rectum, pseudopiloric metaplasia in the terminal ileum and left colic segment); and composition of the inflammatory infiltrate in the lamina propria (lymphoplasmacytic and granulocytic, with or without eosinophils) and its distribution in the segments examined (homogeneous vs. inhomogeneous). Other minimal lesions, not always present, include the presence of eversion/ulceration and non-necrotizing microgranulomas. The presence of granulocytes within the glandular epithelium (cryptitis) and/or within the lumen of the glands (cryptic pseudoabscesses) identifies disease in the active phase. Some of the minimal lesions, such as morphologic changes in the glands and lymphomonocytic infiltration of the lamina propria, require grading into mild (lesions present in <30% of biopsies), moderate (lesions present in 30% to 60% of biopsies), and severe (lesions present in >60% of biopsies) [34,35].

There is strong evidence implicating the gut microbiota in IBD pathogenesis. IBD preferentially affects sites of high microbial density (e.g., distal ileum and colon) while sparing regions with low microbial load [36]. Dysbiosis, altered composition, diversity, and function of commensal microbial communities appears central, with reductions in bacterial diversity, mainly butyrate-producing and mucin-degrading species, and expansion of taxa with pro-inflammatory potential. Whether these alterations are causal or consequential remains unclear.

Epidemiologically, IBD was once considered to be largely a disease of industrialized Western nations, following a marked North–South gradient. Incidence has since plateaued in the West but is rising in newly industrialized regions, paralleling improvements in hygiene and lifestyle. According to the “hygiene hypothesis,” reduced microbial exposure early in life impairs immune tolerance, predisposing to inflammatory disease. Supporting this, antibiotic use, particularly during childhood, has been associated with increased IBD risk [37]. From the genetic point of view, over the past 25 years, more than 200 genetic loci have been linked to IBD [38], many involved in immune regulation and host–microbe interactions.

Together, these findings underscore the multifactorial pathogenesis of IBD, where genetic susceptibility, microbial perturbations, and environmental factors converge to drive chronic intestinal inflammation [39].

### 3.1. FMT in Ulcerative Colitis

Most studies that used FMT in IBD focused on UC. The first randomized controlled trial (RCT), by Rossen et al. [6], investigated clinical and endoscopic outcomes in 48 patients with mild-to-moderate UC who received FMT via nasoduodenal tube. No significant differences were observed compared to placebo, although responders showed increased donor microbiota diversity.

In Moayyedi et al. [7], 75 patients with active UC without infectious diarrhea were randomized to FMT or placebo. Clinical remission occurred in 24% of FMT recipients versus 5% in the placebo group. Microbiota profiling indicated that a higher abundance of *Lachnospiraceae* in donor stool was associated with treatment response, raising the hypothesis of a “superdonor”.

Paramsothy et al. [8] evaluated an intensive multidonor FMT regimen in 85 patients with active UC. Delivery was via colonoscopy in 85 patients, followed by enemas, 5 days a week for 8 weeks. Clinical and endoscopic remission occurred in 27% of the FMT group versus 8% of the placebo. Microbial diversity increased post-FMT, while the presence of *Fusobacterium* spp. was linked to non-response.

In Costello et al. [9], 73 adults with active UC received colonoscopic FMT followed by two enemas over the first week. At eight weeks, remission was achieved in 31% of the FMT group compared with 8% of placebo; 42% of these subjects maintained remission at 12 months.

The role of FMT in maintaining remission has also been examined. In a cohort of 61 Indian UC patients in clinical remission [10], colonic infusion of FMT or placebo was administered every eight weeks for 48 weeks. Clinical remission was maintained in 87.1% of the FMT group versus 66.7% of placebo; endoscopic remission in 58.1% versus 26.7%; and histological remission in 45.2% versus 16.7%.

Crothers et al. [11] combined colonoscopic FMT with encapsulated FMT in 12 patients with mild-to-moderate UC over 12 weeks. Although statistical significance was not reached, the study highlighted the potential of combination delivery systems.

In a subsequent placebo-controlled trial, Haifer et al. [12] assessed oral lyophilized FMT in 35 patients with active UC, following a two-week antibiotic pre-treatment. After eight weeks, corticosteroid-free remission with endoscopic response was achieved in 53% of the FMT group versus 15% of placebo, supporting the feasibility of oral formulations.

To enhance engraftment, a trial tested rationally selected donors with or without budesonide pre-treatment in active UC patients [13]. Donor-dependent effects were observed, but budesonide did not significantly improve engraftment or clinical outcomes.

A recent study evaluated the repeated administration of frozen oral FMT capsules or sterile fecal filtrates from multiple donors in mild-to-moderate UC, with clinical remission at week 12 as the primary endpoint. Early data suggest that metabolites in fecal filtrates may play a role in efficacy [14].

A systematic review [40] confirmed that FMT is effective in mild-to-moderate UC without increasing adverse events, supporting its role as a potential therapeutic option in this subgroup.

Conversely, a meta-analysis of six RCTs in patients with refractory UC found that FMT did not significantly improve clinical or endoscopic remission rates, suggesting limited efficacy in this patient population despite a positive safety profile [41].

Preliminary evidence has also emerged regarding the use of FMT administered via enema in patients with refractory ulcerative proctitis (UP). In one study including 30 participants with UP, the treatment was well tolerated and demonstrated efficacy in inducing clinical remission [15].

However, the analyzed studies collectively indicate that further prospective trials are required to optimize FMT formulation and dosing, as well as to better characterize its safety profile in UC.

### 3.2. FMT in Crohn’s Disease

Experience of FMT for CD is more limited, although its use has shown potential as an effective treatment option. The first pilot RCT, by Sokol et al. [16], enrolled 17 patients with colonic or ileocolonic CD who received colonic FMT or sham. The primary endpoint, donor microbiota colonization at six weeks, was not achieved. However, steroid-free remission was numerically higher in the FMT group at week 10, suggesting potential benefits, particularly in colonic CD rather than penetrating disease.

Subsequent data are mainly drawn from cohort studies. A meta-analysis by Zhou et al. [42] (12 studies; 228 patients) reported remission in 57% of adults with active CD within 2–4 weeks of FMT, along with significant reductions in Crohn’s Disease Activity Index (CDAI) scores after 4–8 weeks. Subgroup analyses found no major differences between FMT methods, except that antibiotic pre-treatment appeared beneficial.

A double-blind, placebo-controlled trial conducted at three Canadian academic centers randomized 21 and 13 patients to the FMT and placebo groups, respectively. Although both groups showed statistically significant improvements in health-related quality of life, FMT was not effective in inducing clinical or endoscopic remission in these patients with CD [17].

Recently, Chukhlovin et al. [18] observed 15 patients with CD, with situations ranging from remission to high activity of disease, who received FMT standard and via oral capsules administered for two days. A complete clinical response to FMT was observed in 10 of 15 patients.

Overall, the published evidence suggests that FMT may offer short-term benefits in active CD. However, more placebo-controlled RCTs with longer follow-up are required to establish its efficacy and durability.

## 4. FMT in Immune Checkpoint Inhibitors Induced Colitis

In recent years, FMT has emerged as a promising therapeutic tool in oncology, both for modulating drug response and for mitigating treatment-related toxicities. ICIs, approved for several malignancies, act by enhancing lymphocyte activity against neoplastic cells and preventing tumor immune escape [43]. Their main molecular targets include CTLA-4 (ipilimumab, tremelimumab), PD-1 (nivolumab, pembrolizumab, cemiplimab, dostarlimab), PD-L1 (atezolizumab, avelumab, durvalumab) and LAG-3 (relatlimab) [44].

Although highly effective, ICI can trigger immune-related adverse events, particularly diarrhea and colitis, which often mimic IBD both endoscopically and histologically. Reported histological patterns include eosinophilic gastroenterocolitis, apoptotic colitis, IBD-like lesions, microscopic colitis (lymphocytic or collagenous), and ischemic-like changes [45].

The pathophysiology of ICI-iC remains incompletely understood, but evidence points to altered cytokine networks and gut microbial dysbiosis [46,47]. Elevated IL-6 levels have been associated with disease activity [48,49,50,51], while a higher baseline abundance of *Bacteroidetes* has been linked to reduced risk [52,53,54]. Conversely, antibiotic exposure appears to worsen severity [55,56]. In preclinical models, *Bifidobacterium* enhanced IL-10-mediated regulatory T-cell function, reducing colitis severity [56,57], whereas *Lactobacillus* supplementation ameliorated disease [58]. These findings support the rationale for microbiota-based therapies such as FMT.

The first use of FMT for ICI-iC was reported in 2018 [19] in patients with steroid-refractory disease, and FMT is now included in guidelines as a potential option for severe, treatment-resistant cases [59].

In 2021, Davar. et al. [20] conducted a clinical trial to evaluate the safety and efficacy of responder-derived FMT in combination with anti-PD-1 therapy in patients with PD-1-refractory melanoma. The combination was well tolerated and provided clinical benefit in 6 of 15 patients, inducing rapid and durable changes in the gut microbiota. The six responders exhibited an increased abundance of taxa previously associated with response to anti-PD-1 therapy, enhanced CD8+ T cell activation, and a reduced frequency of interleukin-8-expressing myeloid cells.

During the same year, Baruch et al. [21] administered FMT in combination with a reinduction of anti-PD-1 immunotherapy in 10 patients with anti-PD-1-refractory metastatic melanoma. Clinical responses were observed in three patients, including two partial responses and one complete response. FMT treatment was associated with favorable changes in immune cell populations and gene expression in both the gut and tumor. Together, these studies demonstrate that FMT from immunotherapy responders can help overcome resistance to anti-PD-1 therapy in patients with melanoma.

Recent studies by Halsey et al. [22] and Elkrief et al. [23] and have strengthened this evidence. In the first [22], of 12 patients with ICI-iC refractory to corticosteroids and biologics (infliximab or vedolizumab), FMT induced symptom improvement in 83%. With repeat FMT when required, 92% achieved clinical remission, and 42% achieved combined endoscopic and histological remission. Responders showed increased microbiota α-diversity with enrichment in *Collinsella* and *Bifidobacterium*. Histological responders had reduced CD8+ T-cell infiltration. In 2024, Elkrief et al. [23] treated five patients with steroid and biologic-refractory ICI-iC with FMT. Four achieved clinical improvement; two relapsed after antibiotic therapy but improved after a second FMT.

In 2024, Kim et al. [24] conducted a clinical trial combining an anti-PD-1 inhibitor with FMT derived from patients who had previously responded to anti-PD-1 therapy. The study enrolled 13 patients with advanced solid tumors refractory to anti-PD-1 treatment. FMT, administered via colonoscopy, induced sustained changes in the gut microbiota and provided clinical benefit in 6 of 13 patients, including one partial response and five cases of stable disease. These data, although preliminary, highlight the therapeutic feasibility of FMT in steroid-refractory ICI-iC.

## 5. FMT in Gastrointestinal Graft-Versus-Host Disease

Graft-versus-host disease is a serious and frequent complication in transplantation. GVHD occurs when the T cells in the graft recognize the recipient as foreign tissue and initiate an immune response against the host. GVHD is a major complication of allogeneic stem cell transplantation (allo-SCT), affecting up to 50% of recipients. Any part of the body can be affected. The most common targets are the skin, liver and gastrointestinal tract. GVHD can be acute (within 3 months of transplant or upon withdrawal of immunosuppression) or chronic (within the first year) [60]. When the GI tract is affected, it is referred to as GI-GVHD. Symptoms include diarrhea, abdominal pain, nausea, vomiting, dysphagia, and anemia, with the lower intestine most frequently involved. Diarrhea is the predominant manifestation. Diagnosis relies on laboratory and stool tests, imaging (ultrasound, CT, MRI), and endoscopy with biopsies [60].

Histologically, an apoptotic pattern, even if not pathognomonic, is very suggestive of acute GVHD, whereas an IBD-like pattern and apoptotic pattern are most frequently observed in chronic GVHD. Moreover, an eosinophilic-rich pattern is shared with both GVHD and mycophenolate mofetil toxicity, leading to the need for a clinical correlation for a differential diagnosis [61,62].

The pathogenesis of GI-GVHD is unclear but likely multifactorial, with growing evidence implicating the gut microbiota. Dysbiosis can occur early on due to chemotherapy, radiation, antibiotics, malnutrition, or transplantation itself. Microbial diversity correlates strongly with outcomes: in one study, the 3-year survival rates were 36%, 60%, and 67% in patients with low, medium, and high post-transplant microbial diversity, respectively [63].

The first-line treatment for GVHD relies on corticosteroids, though responses are often incomplete. Second-line therapies remain non-standardized. In this context, FMT has gained attention as a potential adjunctive therapy [64].

The first study investigating FMT in steroid-resistant acute GVHD was conducted in Japan in 2016 [25]. Four patients received FMT via nasoduodenal tube. All four responded to treatment, with three achieving complete remission and one achieving partial remission. No serious adverse events were reported. Despite the small sample size, this study represented a milestone in the management of GI-GVHD.

In 2021, Spindelboeck et al. [26] conducted a controlled study in nine patients with severe, treatment-refractory GI-GVHD. Four patients responded to FMT administered via colonoscopy, which was associated with improved survival. Responders exhibited gut microbiota features more closely resembling donor profiles, including higher alpha and beta diversity and enrichment of anaerobic butyrate-producing bacteria. Antibiotic exposure was identified as a major factor contributing to FMT failure. These findings suggest that FMT can restore microbial diversity in GI-GVHD patients.

A phase I/II clinical trial of FMT for steroid-refractory grade IV GI-GVHD included 41 patients (23 FMT, 18 control) [27]. The FMT group exhibited higher clinical remission and overall survival, with lower mortality, highlighting FMT as a promising therapeutic option.

Liu et al. [28] studied FMT combined with ruxolitinib (a Janus kinase inhibitor) in 21 patients with intestinal steroid-refractory acute GVHD. The overall response rate at day 28 was 71.4%, with responders showing improved gut microbiota composition.

A 2022 systematic review pooled data from six studies and five case reports, including 79 patients [65]. Complete remission occurred in 55.9% and partial remission in 26.5%, yielding an overall response rate of 82.4%. The analysis concluded that FMT is a safe and effective strategy for GVHD management. However, they argued that due to limited patient numbers and the absence of large, randomized trials, FMT cannot yet be considered standard care, though its low toxicity and clinical benefits justify further evaluation in randomized studies.

In 2023, a prospective, international, single-arm phase 2a study assessed pooled allogeneic fecal microbiota (MaaT013) in 52 patients with grade III–IV, steroid-refractory GI-GVHD (24 from the prospective study and 28 from compassionate use) [29]. At day 28, GI-overall response rates were 38% and 58%, respectively. The twelve-month overall survival was 25% in the prospective cohort and 38% in the compassionate cohort. Adverse events were reported, primarily bacteremia and sepsis. Responders showed increased abundance of beneficial bacteria and short-chain fatty acids, whereas non-responders had higher levels of pathogenic bacteria.

Recently, Yang et al. [30] evaluated FMT in 12 patients with refractory chronic GVHD. FMT was delivered via colonoscopy, and the response was assessed at 12 weeks. One patient achieved complete response, and five achieved partial response. Those with refractory GVHD had lower baseline diversity and higher levels of *Escherichia-Shigella* and *Enterobacteriaceae*. Post-FMT, microbial diversity increased, short-chain fatty acid levels improved, and pathogenic taxa decreased. Recently, Chukhlovin et al. [18] also observed 12 patients with experience of GVHD, after FMT via oral capsules administered for two days. Complete clinical response to FMT was observed in five patients.

Collectively, donor FMT appears to be a safe and feasible adjunctive strategy during both first- and later-line therapy, with potential utility in managing treatment-related colitis [66]. However, its efficacy and its role in preventing immune-related adverse events remain to be clarified in RCTs, as does its potential to improve gastrointestinal symptoms, overall survival, and quality of life.

## 6. Discussion

The gut microbiota and the immune system are intricately interconnected, and their crosstalk plays a central role in bowel diseases, including ICI-iC, GVHD, and IBD. Despite arising from distinct triggers, these conditions exhibit pathophysiological, morphological, and microscopic features that contribute to disease onset and progression.

Importantly, a shared pathogenic axis emerges from gut microbiota dysbiosis, characterized by epithelial barrier disruption, excessive innate immune overactivation, and dysregulated adaptive immune responses that are common to these conditions. These mechanisms ultimately lead to similar patterns of mucosal injury, impaired tissue repair, and sustained disease progression.

Notably, dysbiosis, often induced by chemotherapy, radiotherapy, antibiotics, or the disease itself, disrupts key microbial functions required for immune homeostasis, barrier integrity, and the regulation of intestinal inflammation.

Conversely, FMT is increasingly being explored as a therapeutic strategy across immune-mediated gastrointestinal disorders. By modulating the gut microbial community, rebalancing T-cell subsets, enhancing short-chain fatty acid (SCFA) production, and regulating systemic inflammation, FMT has the potential to restore intestinal homeostasis, attenuate mucosal injury, and improve clinical outcomes in IBD, ICI-iC, and GI-GVHD.

In IBD, and particularly UC, FMT has been shown to induce remission [6,8,9,12,18]. However, efficacy varies depending on donor microbial composition [7], route of administration, and patient characteristics. Clinical responses remain inconsistent [40], and the precise mechanisms through which FMT attenuates intestinal inflammation are still being investigated.

The expanding use of ICIs for malignancies has led to a rise in immune-mediated colitis, a potentially severe complication that can limit cancer therapy. FMT offers a potential therapeutic approach by restoring microbial balance and regulating immune responses, thereby promoting remission without compromising the antitumor efficacy of ICIs [20,21,22,23,24]. Nevertheless, available evidence is limited, and randomized controlled trials are required to confirm efficacy and safety in this setting.

Gastrointestinal involvement in GVHD is a frequent and life-threatening complication of allogeneic hematopoietic stem cell transplantation. The gut microbiota shapes donor immune responses against host tissues and contributes to disease severity. FMT has demonstrated promise in steroid-refractory GVHD by supporting microbial recolonization and reducing intestinal inflammation [65]. However, variability in treatment response suggests that efficacy depends on both donor microbiota composition and the recipient’s immune status. Importantly, the risk of opportunistic infections in immunocompromised patients necessitates close clinical monitoring.

FMT shows promise in restoring microbial balance, immune regulation, and epithelial integrity in IBD, ICI-iC, and GI-GVHD. However, several critical knowledge gaps remain. High-quality evidence is largely limited to IBD, while data for ICI-induced colitis and GI-GVHD are mostly derived from case series and open-label studies. Despite encouraging results, variability in clinical response, safety concerns, and the lack of standardized protocols highlight the need for controlled studies to optimize donor selection, dosing, and delivery strategies.

A mechanistic understanding of how FMT restores immune homeostasis, modulates T-cell subsets, or influences short-chain fatty acid production remains incomplete and debated. Optimal donor selection (whether using commercial fecal products, healthy volunteers, or patients with prior response to immunotherapy), donor characteristics (healthy volunteers, own material or patients responders to therapy), dosing (e.g., starting with ~50 g of fecal material), and route of administration (colonoscopy, enema, capsules, etc.) are not well defined. Long-term safety, particularly in immunocompromised subjects, also remains uncertain.

The interpretation of available evidence is further complicated by heterogeneity in study design (case series versus double-blind, randomized, placebo-controlled trials), primary outcomes (clinical remission, maintenance of remission, or FMT salvage therapy), microbial composition, and treatment protocols. Small sample sizes, variable clinical responses, and biases inherent to non-randomized studies limit the generalizability of findings and hinder robust conclusions regarding efficacy and safety.

Addressing these gaps will require well-designed, controlled studies to standardize FMT protocols, clarify mechanisms of action, and identify key microbial taxa responsible for therapeutic benefit. Expanding randomized trials to ICI-iC and GI-GVHD is essential, alongside an exploration of precision microbiome therapies.

Although generally well tolerated, FMT carries potential risks such as pathogen transmission and uncertain long-term effects, particularly in immunocompromised populations.

Future research should focus on clarifying the mechanisms by which FMT restores immune homeostasis, identifying key bacterial taxa responsible for therapeutic benefit, and establishing standardized treatment procedures. Well-designed randomized trials will be essential to optimize efficacy and ensure safety.

Looking forward, live biotherapeutic products (LBPs) could represent a promising and more controlled alternative or complement to FMT [67]. By using defined bacterial strains, LBPs may restore microbial balance and modulate immune and epithelial functions while potentially reducing risks associated with donor variability and pathogen transmission. However, clinical data in IBD, ICI-induced colitis, and GI-GVHD are scarce, and optimal strains, dosing regimens, and long-term safety profiles are still undefined [68]. However, there is also evidence that single- and multiple-strain probiotics and synbiotics may help with some gastrointestinal disorders. However, concerns about data quality, safety for vulnerable populations, and regulatory issues prevent widespread use.

Looking ahead, live biotherapeutic products (LBPs) may represent a controlled alternative or complement to FMT [67]. By using defined bacterial strains, LBPs can restore microbial balance and modulate immune and epithelial functions, potentially reducing risks related to donor variability and pathogen transmission. However, clinical evidence in IBD, ICI-induced colitis, and GI-GVHD are still limited, and optimal strains, dosing regimens, and long-term safety remain undefined. Similarly, single- and multi-strain probiotics or synbiotics may offer some benefit in gastrointestinal disorders [68], but concerns regarding data quality, safety in vulnerable populations, and regulatory challenges currently limit their broad application.

## 7. Conclusions

Overall, FMT represents a rapidly evolving field with the potential to induce remission in IBD, mitigate immune-related adverse events during cancer therapy, and improve outcomes following allogeneic stem cell transplantation. However, the collective findings emphasize the importance of conducting mechanistic studies to clarify the microbiome–host interactions that influence responsiveness to FMT in these conditions.

With further refinement and validation through controlled trials, FMT could become an important adjunctive therapy for managing immune-mediated gastrointestinal diseases.

## Figures and Tables

**Table 1:** Summary of studies investigating the integration of fecal microbiota transplantations (FMT) with Crohn’s disease (CD), ulcerative colitis (UC), “gastrointestinal graft-versus-host disease (GI-GVHD), and immune checkpoint inhibitor-induced colitis (ICI-iC); * urothelial carcinoma and ** prostate cancer.

Authors	Patient Population	Study Design	Number of Recipients	FMT Adminitration/Protocol	Donor Type	Primary Outcome	Major Findings
Rossen et al. [6]	Mild to moderate active UC	Double-blind, randomised, placebo-controlled	50	Nasoduodenal tube	Own FMT VS healthy donors FMT	Clinical remission	No statistical difference
Moayyedi et al. [7]	Active UC without infectious diarrhea	Double-blind, randomised, placebo-controlled	75	Via enema	Plalcebo VS healthy donors FMT	Clinical remission	Clinical remission in the FMT group
Paramsothy et al. [8]	Active UC	Double-blind, randomised, placebo-controlled	85	Colonoscopy followed by enema	Plalcebo VS healthy multidonors FMT	Clinical remission	Clinical remission in the FMT group
Costello et al. [9]	Mild to moderate active UC	Double-blind, randomised, placebo-controlled	73	Colonoscopy followed by enema	Own FMT VS healthy donors FMT	Clinical remission	Clinical remission in the FMT group
Sood et al. [10]	Clinical remission UC	Double-blind, randomised, placebo-controlled	61	Colonoscopy	Plalcebo VS healthy donors FMT	Maintain clinical remission	Clinical remission in the FMT group
Crothers et al. [11]	Active UC	Double-blind, randomised, placebo-controlled	12	Oral administration of frozen encapsulated FMT	Plalcebo VS healthy donors cFMT	Clinical remission	Safety
Haifer et al. [12]	Active UC	Double-blind, randomised, placebo-controlled	35	Oral administration of lyophilised encapsulated FMT	Plalcebo VS healthy donors lyophilised FMT	Clinical remission with endoscopic remission or response	Clinical remission in the FMT group
van Lingen et al. [13]	Active mild to moderate disease	Double-blind, randomised, placebo-controlled	24	Colonoscopy	Plalcebo VS healthy donors FMT	Engraftment of donor microbiota after FMT	Pretreatment with budesonide did not significantly influence engraftment or clinical response
Stallmach et al. [14]	Mild to moderate active UC	Double-blind, randomised, placebo-controlled	174	Oral administration of frozen encapsulated FMT	Placebo VS sterile FMT VS healthy donors FMT	Clinical remission	Microbial and immunologic changes after FMT
Raja et al. [15]	Resistant ulcerative proctitis (UP) or distal UC	Double-blind, randomised, placebo-controlled	30	Via enema	Single-donor FMT	Safety and tolerability of FMT therapy	FMT enema was well tolerated and efficacy in inducing clinical remission
							
Sokol et al. [16]	CD in clinical remission	Single-blind, randomized, placebo-controlled	17	Colonoscopy	Plalcebo VS healthy donors FMT	Clinical remission	No statistical difference
Kao et al. [17]	Mild-to-moderate CD	Double-blind, randomised, placebo-controlled	44	Colonoscopy followed by capsules	Plalcebo VS healthy donors FMT	Clinical and endoscopic remission	No statistical difference
Chukhlovin et al. [18]	Remission-to-high activity CD	Case series	15	Oral administration of frozen encapsulated FMT	Healthy donors FMT	Clinical response	Ten patients with complete clinical response
							
Wang et al. [19]	* Urothelial carcinoma refractory to standard chemotherapy threated with CTLA-4 and PD-1; ** Prostate cancer refractory to chemotherapy and hormonal therapy who received two doses of ipilimumab	Case series	2	* Colonoscopy single dose; ** two doses of FMT	Single healthy donor FMT	Complete resolution of clinical symptoms following treatment with FMT	*Complete resolution; **Complete resolution after the second FMT
Davar et al. [20]	Melanoma patients refractory to anti–PD-1 therapy	Case series	15	Colonoscopy	FMT from anti-PD-1 responder and anti–PD-1 therapy	FMT salvage therapy	Six of 15 patients with clinical benefit
Baruch et al. [21]	Melanoma patients refractory to anti–PD-1 therapy	Case series	10	Colonoscopy	FMT from anti-PD-1 responder and anti–PD-1 therapy	FMT salvage therapy	Three of 10 patients with clinical benefit
Halsey et al. [22]	Different cancers treated with CTLA-4 and PD-1 or combined drugs	Case series	12	Colonoscopy	FMT from healthy donors	FMT salvage therapy	Ten patients achieved symptom improvement after FMT, including seven patients who had a complete clinical response. Three patients required repeat FMT, one of which had response. At the end of the study period, 92% achieved immune-mediated colitis clinical remission
Elkrief et al. [23]	Different cancers treated with CTLA-4 and PD-1 or combined drugs	Case series	5	Colonoscopy	FMT from healthy donors	FMT salvage therapy	Four of 5 patients exhibited improvement in immune checkpoint inhibitors Colitis symptoms following FMT
Kim et al. [24]	Different cancers treated with PD-1	Case series	13	Colonoscopy	FMT from anti-PD-1 responder	FMT salvage therapy	Six of 13 patients acghieved clinical benefits
							
Kakihana et al. [25]	GI-GVHD	Case series	4	Nasoduodenal tube	FMT from healthy donors	FMT salvage therapy	Three achieved complete responses; one partial responses
Spindelboeck et al. [26]	GI-GVHD	Case series	9	Colonoscopy	FMT from healthy donors	FMT salvage therapy	Four patients with clinical response and was observed a change in immune cell patterns
Zhao et al. [27]	GI-GVHD	Open-label, non-randomized	41	Nasoduodenal tube	None FMT VS healthy donors FMT	Clinical remission	Clinical remission in the FMT group
Liu et al. [28]	GI-GVHD threated with Ruxolitinib	Case series	21	Oral administration of frozen encapsulated FMT	FMT from healthy donors	FMT salvage therapy	Fifteen patients with clinical response, 10 of them with complete response
Malard et al. [29]	GI-GVHD	Case series	76	Via rectal catheter	Pooled allogeneic faecal microbiota MaaT013 at leat one dose	FMT salvage therapy	Thirtynine patients with clinical response, 22 of them with complete response
Yang et al. [30]	GI-GVHD	Case series	12	Colonoscopy	FMT from healthy donors	FMT salvage therapy	Six patients with clinical response, 1 of them with complete response
Chukhlovin et al. [18]	GI-GVHD	Case series	12	Oral administration of frozen encapsulated FMT	Healthy donors FMT	Clinical response	Five patients with complete clinical response

## Abbreviations

The following abbreviations are used in this manuscript:

FMT	Fecal Microbiota Transplantation
IBD	Inflammatory Bowel Disease
CD	Crohn’s Disease
UC	Ulcerative Colitis
ICI-iC	Immune Checkpoint Inhibitor-Induced Colitis
GI-GVHD	Gastrointestinal Graft-Versus-Host Disease
RCT	Randomized Controlled Trial
